# Neuromedin U receptor 2 agonists suppress cocaine self-administration in male rats

**DOI:** 10.21203/rs.3.rs-11000092/v1

**Published:** 2026-10-05

**Authors:** Vena Martinez, Hong Sun, Cun Zhang, Robert Fox, Jia Zhou, Kathryn Cunningham, Jonathan Hommel

**Affiliations:** The University of Texas Medical Branch at Galveston

## Abstract

**Rationale:**

Cocaine use disorder (**CUD**) remains without an FDA-approved pharmacotherapy, highlighting the need for novel mechanistically distinct targets. Prior work implicates neuromedin U (**NMU**) and its receptor NMU receptor 2 (**NMUR2**) in cocaine-directed behavior, but NMUR2 peptide-based approaches have limited translational utility.

**Objectives:**

Here, we tested whether the previously characterized NMUR2-active small molecules NY0116 and NY0128 reduce cocaine self-administration under a progressive ratio schedule in male rats. Because reduced progressive ratio responding can also reflect broader behavioral disruption, we examined response latency, inactive lever responding, and locomotor activity.

**Methods:**

Rats were trained to self-administer intravenous cocaine (0.25 mg/kg/infusion) and tested after systemic administration of NY0116 or NY0128.

**Results:**

Both compounds reduced cocaine-maintained responding at the highest dose tested, including reductions in breakpoint and active lever responding. Lever discrimination was not significantly altered at the effective dose, arguing against loss of task discrimination as the explanation for reduced cocaine intake. Individual-animal analyses showed no significant correlation between levels of locomotor activity and cocaine infusions during NY0128 test sessions, indicating that locomotor output did not account for individual differences in cocaine intake.

**Conclusions:**

These findings demonstrate that NY0116 and NY0128 are effective in reducing cocaine self-administration in male rats, providing strong evidence that NMUR2 is a promising target for developing new CUD pharmacotherapies.

## INTRODUCTION

Cocaine is the prevalent drug of choice for 3.0 million persons in the U.S. with cocaine use disorder (**CUD**) diagnosed in 1.3 million people in 2024 [1], contributing to the global burden of drug-related morbidity and mortality [2]. Despite decades of research, no CUD pharmacotherapy has been approved by the U.S. Food and Drug Administration. One emerging target is the neuromedin U (**NMU**) signaling system and its predominantly central receptor NMU receptor 2 (**NMUR2**) [3, 4], which has been implicated in the regulation of motivated behaviors [5-7].

Our previous work identified NMUR2 signaling as a negative regulator of the rewarding effects of cocaine [8, 9]. Systemic administration of the endogenous peptide NMU reduced breakpoint for cocaine under progressive ratio (**PR**) schedule, whereas knockdown of NMUR2 produced the opposite behavioral phenotype, supporting a functional role for NMUR2 activation in limiting cocaine-maintained responding [8]. Collectively, these findings established NMUR2 as a candidate pharmacological target for CUD. However, these studies relied on the endogenous peptide agonist. Whether pharmacological activation of NMUR2 with a chemically distinct, systemically active small-molecule agonist similarly reduces cocaine intake has not been determined.

To address this question, we recently developed and characterized the small-molecule NMUR2 agonists NY0116 and NY0128. These compounds exhibit NMUR2-dependent calcium and cAMP signaling *in vitro*, demonstrate measurable systemic and brain exposure following peripheral administration, and produce biological effects consistent with NMUR2 activation *in vivo* [10]. Together, these findings establish NY0116 and NY0128 as pharmacologically validated NMUR2 agonists and provide an opportunity to reproduce the previously observed effects of the endogenously administered peptide on cocaine-directed behavior. Demonstrating efficacy with chemically distinct agonists would provide pharmacological proof-of-concept that NMUR2 is amenable to small-molecule modulation in the context of cocaine reinforcement.

Progressive ratio schedules of intravenous self-administration are widely used to evaluate the reinforcing efficacy of drugs by requiring progressively greater effort to obtain successive infusions, with breakpoint serving as an index of maximum effort an animal will expend to obtain cocaine [11]. Interpretation of reductions in cocaine-maintained responding requires consideration of potential nonspecific behavioral effects. Accordingly, complementary behavioral measures collected during PR testing, including inactive lever responding, response latency, lever discrimination, and locomotor activity, are key to determining whether changes in cocaine self-administration are consistent with reduced reinforcing effects of cocaine rather than generalized disruption of operant performance.

In the present study, we tested whether systemic administration of NY0116 and NY0128 decreases cocaine self-administration under a PR schedule in male rats. We further evaluated inactive lever responding, response latency, lever discrimination, and concurrent locomotor activity to assess the behavioral specificity of these effects. Together, these experiments extend previous observations obtained with the endogenous NMU peptide by establishing whether pharmacological activation of NMUR2 with systemically active small molecules is sufficient to reduce the reinforcing efficacy of cocaine while providing pharmacological proof-of-concept for future pharmacological development targeting the NMU/NMUR2 signaling system.

## MATERIALS AND METHODS

### Animals

Male Sprague-Dawley rats (n = 24; Envigo, Houston, TX, USA) weighed approximately 175 g on arrival. Rats were housed in pairs without additional enrichment and had *ad libitum* access to standard laboratory chow and water except during behavioral testing. The colony was maintained at 71°F and 30–50% relative humidity on a 12-h light/dark cycle, with lights on from 06:00 to 18:00. All procedures were conducted in accordance with the National Institutes of Health Guide for the Care and Use of Laboratory Animals and were approved by the University of Texas Medical Branch Institutional Animal Care and Use Committee. All investigators conducting experiments and endpoint analyses were blinded to treatment assignments. Our adherence to rigorous, transparent animal research using the ARRIVE Essential 10 guidelines enhances reproducibility, thereby creating a solid foundation for future research [12].

### Drugs

Cocaine hydrochloride was provided by the National Institute on Drug Abuse Drug Supply Program (Research Triangle Park, NC, USA) and dissolved in sterile 0.9% saline. Cocaine was delivered intravenously at 0.25 mg/kg per infusion in a volume of 0.1 mL. NY0116 and NY0128 were synthesized as previously described and had been characterized for NMUR2-linked calcium and cAMP signaling, plasma and brain exposure after systemic administration, and *in vivo* efficacy in feeding and body-composition models [10]. For the present experiments, NY0116 and NY0128 were prepared in sterile 0.9% saline containing 5% dimethyl sulfoxide and 10% Kolliphor and administered subcutaneously at 1 mL/kg. NY0128 was selected for the locomotor analysis demonstrating the lower EC_50_ in the prior compound characterization [10].

### Cocaine Intravenous Self-Administration

Rats were implanted with chronic indwelling jugular catheters connected to a back-mounted access port using procedures described previously [8, 14]. Animals were anesthetized for surgery, and the catheter was advanced into the jugular vein and secured, with the external access port positioned subcutaneously on the back. Rats were allowed at least seven days to recover prior to training in cocaine self-administration. Catheters were maintained and assessed for patency throughout the experiment according to the established laboratory procedures [14, 16]. Animals were removed from analysis when catheter patency could no longer be confirmed.

Self-administration sessions were conducted in standard operant conditioning chambers equipped with two retractable levers and an intravenous infusion pump. Daily sessions lasted 180 min, consistent with the previously published procedure from this laboratory [8, 16-20]. Responding on the active lever produced a cocaine infusion, whereas responses on the inactive lever were recorded but had no programmed consequences. Rats first acquired cocaine self-administration under a fixed-ratio 1 (**FR1**) schedule, in which one active-lever response produced an infusion. The response requirement was then increased to FR5 before animals were transferred to the progressive-ratio schedule. Under the progressive-ratio schedule, the number of responses required for successive infusions increased across the session using the same response progression described previously [21]. Breakpoint was defined as the final ratio completed during the session. Active-lever responses, inactive-lever responses, cocaine infusions, and latency to the first infusion were recorded.

Rats were considered stable when cocaine self-administration varied by less than 10% across three consecutive sessions. Stability was also evaluated across vehicle test days before interpreting compound effects. Separate cohorts were employed to test NY0116 and NY0128. Each cohort initially included 12 rats; the final analyses included 11 rats for NY0116 and 10 rats for NY0128 after loss of catheter patency. The study used a within-subject dose design. Vehicle and NY0116 or NY0128 doses of 3, 10, and 30 mg/kg were administered subcutaneously 1 h after the final cocaine self-administration session, and animals were tested 24 h later. Test doses were separated by at least seven days. Because the compounds have a prolonged pharmacokinetic profile, doses were tested in ascending order to minimize the possibility that residual exposure to a higher dose would influence a subsequent lower-dose test [10].

Locomotor activity was recorded concurrently during cocaine self-administration in the NY0128 cohort using two horizontal photobeams integrated into each operant chamber. Beam interruptions were summed as the measure of locomotor activity. For correlational analyses in individual animals, cocaine infusions and beam breaks during the first 60 min of each vehicle or NY0128 test session were analyzed. This analysis was designed to test the hypothesis that rats with lower locomotor output also obtained fewer cocaine infusions within a given treatment condition.

### Analysis

Statistical analyses were performed using GraphPad Prism version 10. Progressive-ratio outcomes were analyzed separately for NY0116 and NY0128 using repeated-measures one-way analysis of variance or a mixed-effects model when observations were missing because of catheter loss, followed by planned comparisons of each dose with vehicle. Lever discrimination was calculated as active-lever responses divided by the sum of active- and inactive-lever responses and was compared between vehicle, 3 mg/kg, 10 mg/kg, and 30 mg/kg using a paired two-tailed t-test. Pearson correlations were used to evaluate the relationship between cocaine infusions and locomotor activity during the first 60 min of vehicle and NY0128 test sessions. Statistical significance was set at p < 0.05. Data are presented as mean ± SEM with individual-animal values displayed in the figures.

## RESULTS

### Experimental design.

We evaluated NY0116 and NY0128 in separate cohorts of rats that had reached stable cocaine self-administration under a progressive-ratio schedule (Fig. 1). After excluding animals that lost catheter patency, the final analyses included 11 rats in the NY0116 cohort and 10 rats in the NY0128 cohort. Each cohort received vehicle and 3, 10, and 30 mg/kg assigned in a within-subject design, with each treatment given 1 h after the final cocaine self-administration session, responding assessed 24 h later, and at least seven days between compound tests. We dosed 1 h after the session because these compounds penetrate the CNS slowly and clear slowly [10]. In rats given a single injection, plasma NY0116 peaked at 10 h with an elimination half-life of 36.8 h, and NY0128 peaked at 4 h with a half-life of 18.5 h [10]. Dosing 1 h after the session therefore allows central exposure to increase before the test session. Baseline intake stayed steady throughout: the number of infusions did not differ across repeated vehicle tests in either the NY0116 cohort (F3,30 = 1.899, p > 0.05) or the NY0128 cohort (F2,18 = 0.051, p > 0.05). Each set of dose effects was therefore measured against a stable within-subject vehicle baseline.

### NY0116 decreased cocaine-maintained responding.

NY0116 produced significant dose effects across multiple measures: breakpoint (F_3, 30_=5.995, p < 0.05; Fig. 2A), active-lever responses (F_3, 30_=4.501, p < 0.05; Fig. 2B), inactive-lever responses (F_3, 24_=6.204, p = 0.0028; Fig. 2C), and latency to the first infusion (F_3, 30_=3.364, p < 0.05; Fig. 2D). These effects were driven by the highest dose. *Post hoc* comparisons showed that 30 mg/kg lowered breakpoint, active-lever responses, and inactive-lever responses relative to vehicle and lengthened the latency to the first infusion (all p < 0.05), whereas the 3 and 10 mg/kg doses did not differ from vehicle on any of these measures. At the effective dose, NY0116 reduced responding on both levers and delayed the start of cocaine self-administration.

### NY0128 decreased cocaine-maintained responding.

NY0128 also produced significant dose effects on breakpoint (F_3, 24_=20.59, p < 0.05; Fig. 3A), active-lever responses (F_3, 30_=8.546, p < 0.05; Fig. 3B), inactive-lever responses (F_6, 54_=5.161, p < 0.05; Fig. 3C), and latency to the first infusion (F_3, 27_=7.649, p < 0.05; Fig. 3D). As with NY0116, only the highest dose was effective. Relative to vehicle, 30 mg/kg NY0128 lowered breakpoint, active-lever responses, and inactive-lever responses (all p < 0.05), while 3 and 10 mg/kg did not alter these measures. Although the analysis detected latency with 30 mg/kg, none of the individual NY0128 doses differed significantly from vehicle in the planned comparisons. Accordingly, the decrease in progressive-ratio responding at 30 mg/kg was not accompanied by a statistically reliable increase in latency to the first infusion.

### High-dose NMUR2 agonist preserved active-versus-inactive lever discrimination.

Because both compounds reduce active- and inactive-lever responding at 30 mg/kg, we asked whether the effective dose also changed how responses were distributed between the two levers. We calculated the discrimination index, the proportion of total lever responses directed at the active lever, where values near 1 indicate that responding stayed concentrated on the cocaine-associated lever. The index was unchanged at the effective dose as no difference between vehicle and 30 mg/kg NY0116 (p > 0.05; Fig. 4A) nor between vehicle and 30 mg/kg NY0128 (p > 0.05; Fig. 4B) was observed. Group values remained near the top of the scale following both treatments. High-dose treatment therefore lowered the overall amount of lever responding without shifting responding away from the cocaine-associated lever or erasing the distinction between the active and inactive levers.

### Locomotor activity did not covary with cocaine infusions within NY0128 test sessions.

To determine whether individual differences in locomotor activity tracked with cocaine intake, we recorded locomotion simultaneously with self-administration in the NY0128 cohort and correlated beam breaks with cocaine infusions over the first 60 min of each vehicle or NY0128 session (Fig. 5). No correlation reached significance after vehicle (Fig. 5A), mg/kg (Fig. 5B), 10 mg/kg (Fig. 5C), or 30 mg/kg NY0128 (Fig. 5D; all p > 0.05), and the scatterplots did not demonstrate a consistent linear relationship between the two measures. Animals that exhibited less motor activity within a given condition were not reliably the animals that earned fewer cocaine infusions. Within that scope, individual differences in locomotor output did not explain individual differences in cocaine intake during the NY0128 test sessions (Fig. 5E).

### Summary of behavioral effects.

Across two independent cohorts, both NY0116 and NY0128 reduced breakpoint and active-lever responding for cocaine intake at 30 mg/kg, while the lower doses were without significant effects on these primary measures. Both compounds also reduced inactive-lever responding at the effective dose, and NY0116 increased response latency. Despite these accompanying changes, active-versus-inactive lever discrimination remained intact. Taken together, the results point to a reduction in high-effort cocaine-maintained responding, and mark performance-related metrics to weigh in the interpretation of behavioral profiles.

## DISCUSSION

The present study demonstrates that systemic administration of the small-molecule NMUR2 agonists NY0116 and NY0128 decreases cocaine self-administration under a PR schedule of reinforcement. At the highest dose tested, both compounds reduced breakpoint and active-lever responding while preserving discrimination between the active and inactive levers. These findings extend our previous work demonstrating that activation of the NMU/NMUR2 signaling system suppresses cocaine-directed behavior by showing that this behavioral phenotype can be reproduced using systemically active, pharmacologically validated small-molecule agonists. Together, these data provide pharmacological proof-of-concept that NMUR2 signaling can be engaged by non-peptide ligands to reduce the reinforcing efficacy of cocaine.

Our previous work established the biological relevance of NMUR2 signaling in cocaine reinforcement using the endogenous peptide NMU and receptor knockdown approaches [8]. Those studies demonstrated that activation by the endogenous ligand reduced cocaine breakpoint, whereas disruption of NMUR2 signaling produced the opposite behavioral phenotype, identifying NMUR2 as a regulator of cocaine-maintained behavior. The present findings advance that work in an important way. Rather than further defining receptor biology, the current study demonstrates that a chemically distinct class of ligands reproduces the previously established behavioral effects seen with the NMU peptide. This distinction is significant because confidence is strengthened when convergent behavioral outcomes are obtained using independent pharmacological approaches. Although the present work does not constitute a drug development program, we establish that the behavioral effects of NMUR2 activation are not unique to the endogenous peptide and that the receptor is pharmacologically tractable using systemically administered small molecules.

This progression is consistent with a broader trend in addiction pharmacology toward identifying neuromodulatory systems that regulate cocaine-directed behaviors through mechanisms distinct from direct manipulation of monoamine transporters. Numerous neuropeptide systems, including glucagon-like peptide-1, orexin, oxytocin, neurotensin, and nociceptin signaling have emerged as modulators of cocaine reinforcement in preclinical models [14, 15, 22]. Within this context, the present findings further evaluations of NMUR2 signaling as an additional neuropeptide system capable of influencing cocaine-maintained behavior. Importantly, the current study extends this literature beyond endogenous peptide administration by demonstrating that receptor activation can be achieved with pharmacologically characterized small molecules, thereby providing a more experimentally and translationally accessible approach for future investigation. Importantly, this preclinical framework can be extended to human studies that directly assess the reinforcing effects of cocaine. For example, we reported a randomized clinical study of lorcaserin using cocaine versus money progressive ratio choice in individuals with cocaine use disorder. This demonstrates how pharmacological effects on cocaine reinforcement can be evaluated across preclinical and human models [23].

The behavioral effects observed in the present study should be interpreted within the context of PR procedure. PR schedules are widely used to assess the reinforcing efficacy of drugs by increasing the response requirement for successive infusions until responding ceases [11, 13]. Consequently, reductions in breakpoint are generally interpreted as reflecting a reduction in the reinforcing efficacy of cocaine rather than complete suppression of cocaine taking or direct evidence for therapeutic efficacy [8, 13, 15, 18, 21, 23-25]. The present findings therefore indicate that systemic NMUR2 agonism decreases the effort animals are willing to expend to obtain cocaine under conditions of escalating response cost. Whether similar effects would extend to other clinically relevant aspects of CUD, including relapse vulnerability, behavioral economics, cocaine choice, or maintenance of abstinence, remains to be determined.

Interpretation of reduced cocaine-maintained responding also requires consideration of potential nonspecific behavioral effects. Although NY0128 reduced locomotor activity during PR testing, several observations suggest that locomotor suppression alone is unlikely to account for the reduction in cocaine self-administration. Lever discrimination remained intact throughout testing, indicating that animals continued to selectively respond on the cocaine-associated lever despite reduced cocaine intake. Furthermore, locomotor activity was not significantly correlated with cocaine intake across individual animals within any treatment condition. Thus, animals exhibiting greater reductions in locomotor activity were not consistently those exhibiting the greatest reductions in cocaine-maintained responding. These findings are consistent with previous reports [24] and argue against a simple explanation in which reduced cocaine self-administration reflects only diminished motor output, while acknowledging that broader behavioral effects cannot be completely excluded. Accordingly, the present data support the conclusion that NMUR2 agonist decreases cocaine-maintained responding under conditions in which task discrimination is preserved and inter-individual differences in locomotor activity do not explain variability in cocaine intake.

Despite substantial effort over several decades, development of effective pharmacotherapies for CUD has proven challenging, and no medication has received FDA approval for this indication. The NMU/NMUR2 signaling system represents a mechanistically distinct approach by engaging an endogenous neuropeptide pathway rather than directly competing with cocaine at monoamine transporters. It is important to distinguish the behavioral endpoint examined here from broader therapeutic goals. The PR procedure evaluates reinforcing efficacy during ongoing cocaine self-administration and does not model abstinence, relapse prevention, or long-term treatment outcomes. Consequently, the present findings should be viewed as pharmacological proof-of-concept supporting continued investigation of NMUR2 signaling rather than evidence that NMUR2 agonists would constitute effective treatments for CUD.

Several limitations define the scope of the present findings. Only male rats were studied, and compounds were administered in ascending dose because of their pharmacokinetic properties. It is vital to determine if these effects translate to female rats, as sex-dependent differences in neuropeptide signaling and reward-related behavior have been reported [26]. Although concurrent behavioral measures strengthened interpretation of the PR data, additional studies examining cocaine dose-response function, relapse-like behavior, and punishment-resistant cocaine intake are necessary to define the broader behavioral profile of NMUR2 agonists. Likewise, future studies should determine whether these effects generalize across biological sex and evaluate the mechanisms by which NMUR signaling alters cocaine reinforcement.

## CONCLUSIONS

In conclusion, the present study demonstrates that two pharmacologically validated small-molecule NMUR2 agonists decrease the reinforcing efficacy of cocaine under a PR schedule. By reproducing a behavioral phenotype previously observed with the endogenous peptide, these findings provide convergent pharmacological evidence supporting NMUR2 signaling as a regulator of cocaine-maintained behavior. More broadly, they establish pharmacological proof-of-concept that NMUR2 signaling can be engaged by systemically active small molecules, providing a foundation for future mechanistic studies and continued evaluation of NMUR2-directed pharmacological strategies for CUD.

## Figures and Tables

**Figure 1:**
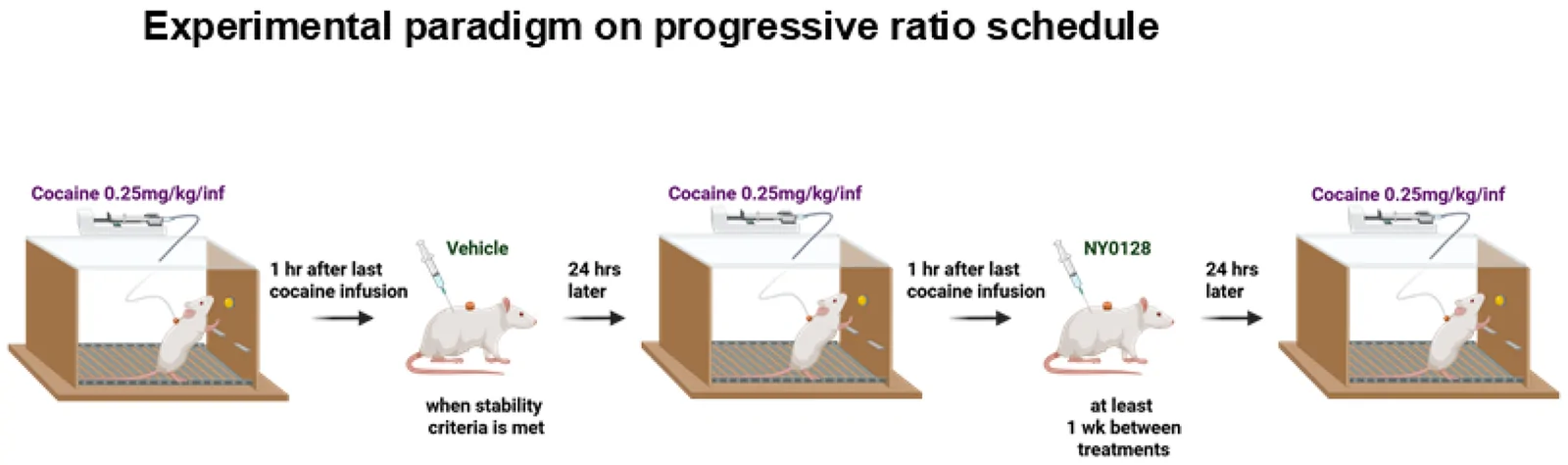
Schematic of experimental paradigm. Animals were trained to self-administer cocaine (0.25 mg/kg/infusion) under a progressive ratio schedule. After reaching stability criteria, animals received vehicle or an NMUR2 agonist one hour after the final cocaine self-administration session and were tested 24 hr later. Doses were tested in ascending order with at least one week between drug tests.

**Figure 2:**
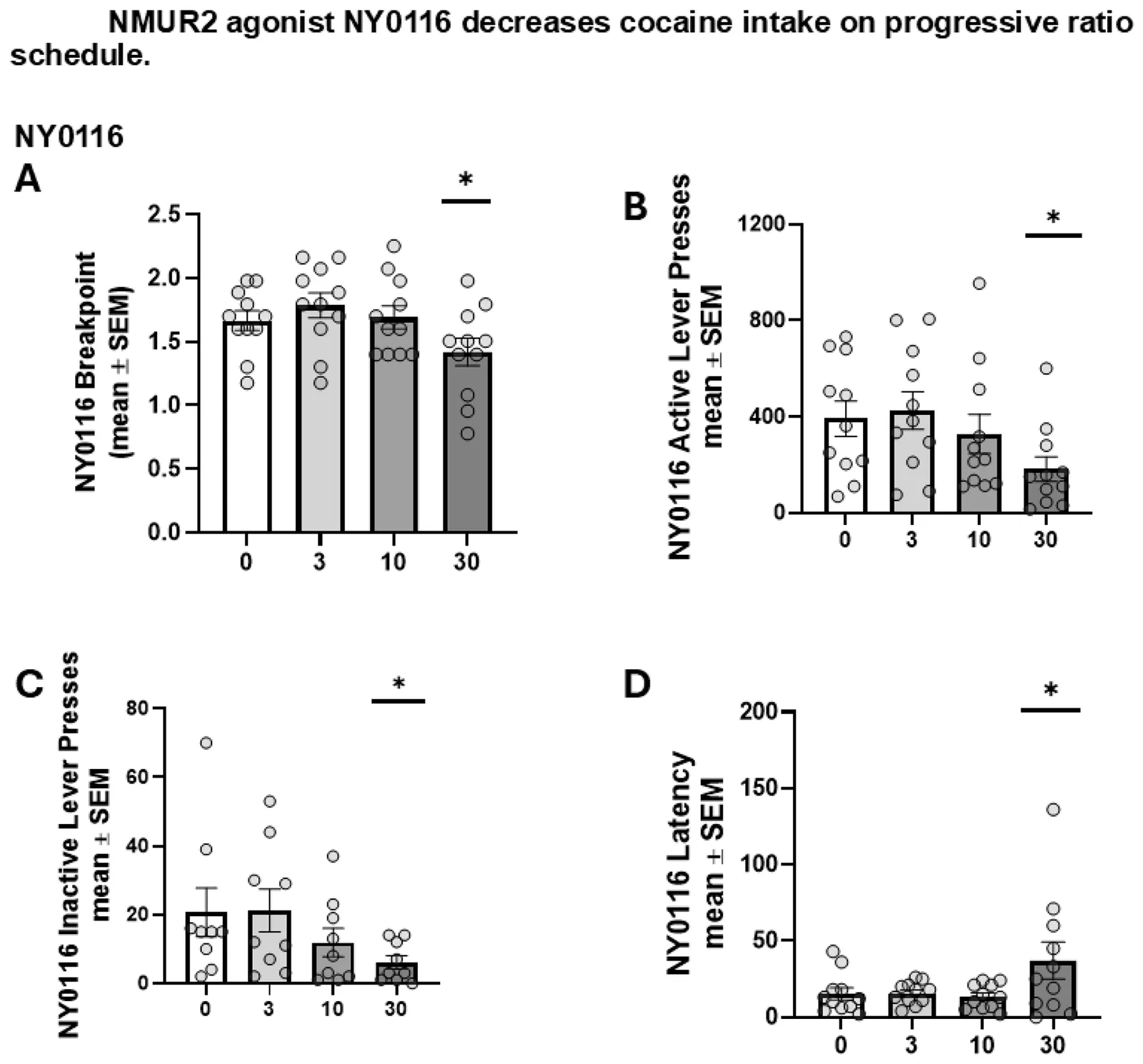
NY0116 decreased cocaine self-administration under a progressive ratio schedule. A-D NY0116 decreased breakpoint, active lever presses, and inactive lever presses, and increased latency to respond (n=11). Bar graphs show mean ± SEM. X-axis indicates dose (mg/kg). *p<0.05 versus vehicle. Repeated-measures one-way ANOVA.

**Figure 3:**
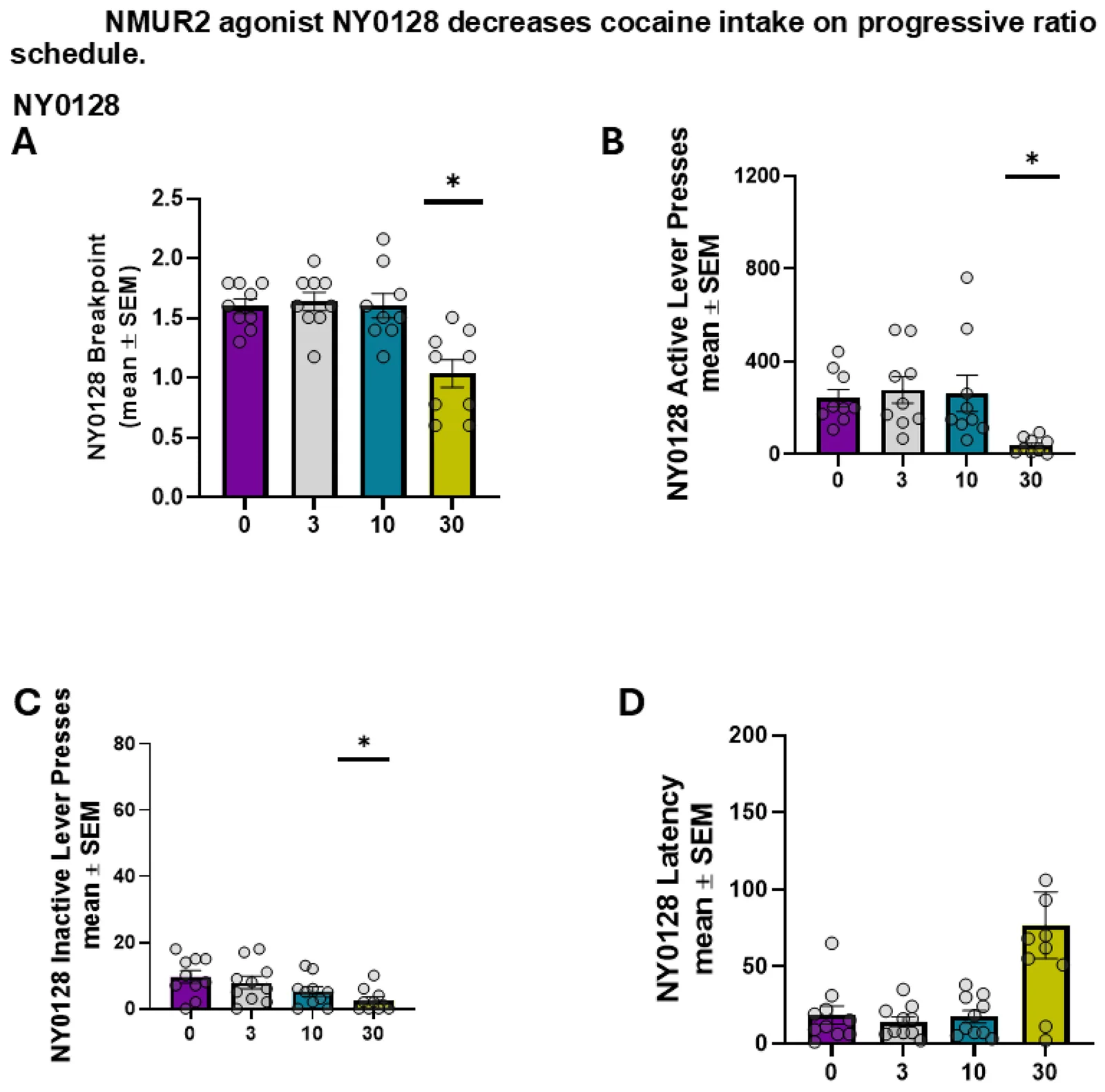
NY0128 decreased cocaine self-administration under a progressive ratio schedule. A-D NY0128 decreased breakpoint, active lever presses, and inactive lever presses, but did not alter latency relative to vehicle (n=10). Bar graphs show mean ± SEM. X-axis indicates dose (mg/kg). *p<0.05 versus vehicle. Repeated-measures one-way ANOVA.

**Figure 4:**
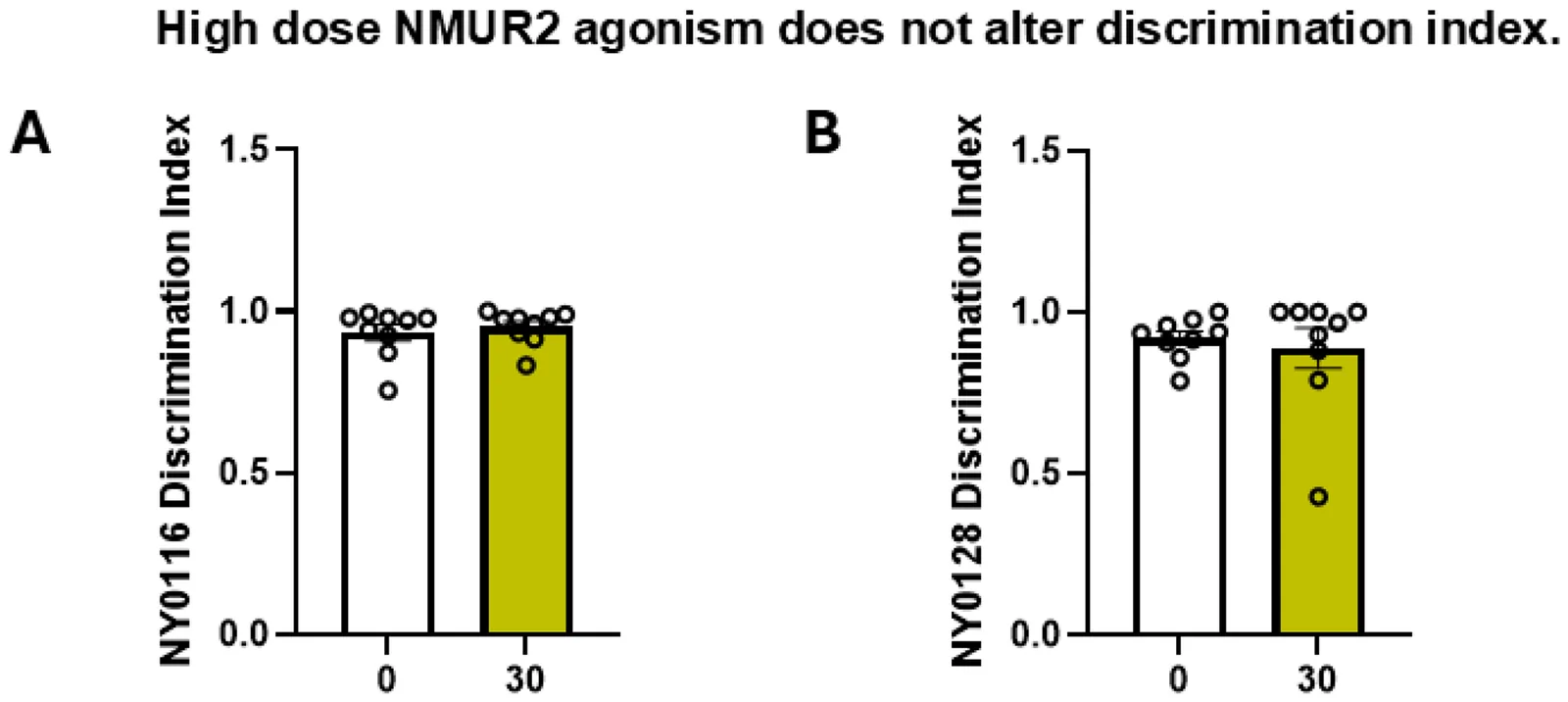
High-dose NMUR2 agonist did not alter lever discrimination. A-B Discrimination index did not differ between vehicle and 30 mg/kg for NY0116 or NY0128. Bar graphs show mean ± SEM. p>0.05. Paired two-tailed t-test.

**Figure 5:**
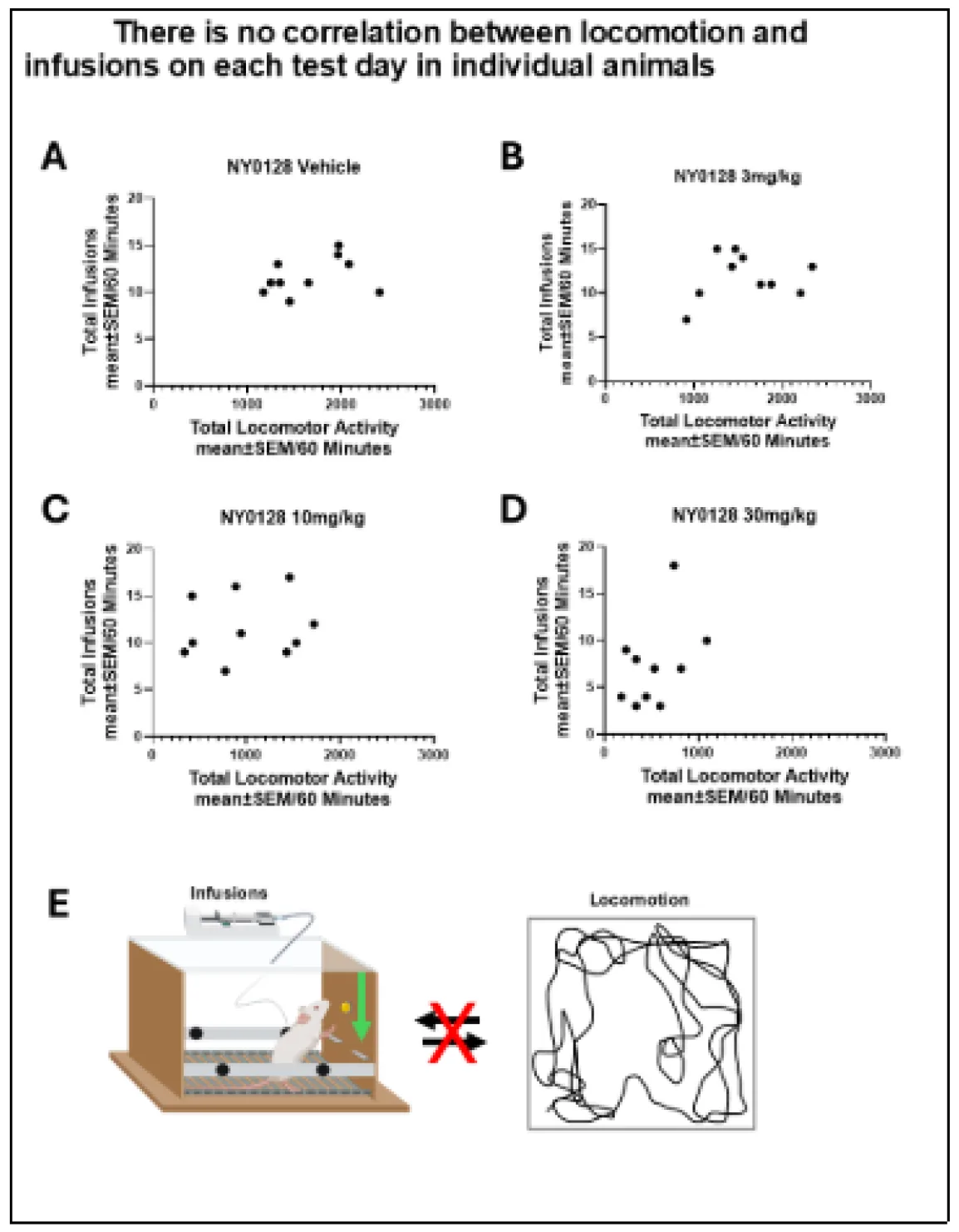
Locomotor activity did not correlate with cocaine infusions during NY0128 test sessions. A-D Pearson correlations between cocaine infusions and locomotor activity (LMA) during vehicle, 3 mg/kg, 10 mg/kg, and 30 mg/kg NY0128 test sessions. Each point represents an individual rat. E Summary schematic indicating that individual-animal LMA did not account for cocaine infusions within test sessions. p>0.05 for all correlations.

## Data Availability

The datasets generated during and/or analyzed during the current study are available from the corresponding author on reasonable request.
